# The use of cell free DNA in the diagnosis of HCC

**DOI:** 10.20517/2394-5079.2019.30

**Published:** 2019-09-23

**Authors:** Bubu A. Banini, Arun J. Sanyal

**Affiliations:** Division of Gastroenterology, Hepatology and Nutrition, Department of Internal Medicine, Virginia Commonwealth University, Richmond, VA 23298, USA.

**Keywords:** Hepatocellular carcinoma, liquid biopsy, cell free nucleic acid, cell free DNA, exosomes, microvesicles, biomarkers

## Abstract

Hepatocellular carcinoma (HCC) is one of the most common malignant tumors worldwide and is associated with high mortality. The currently used methods for diagnosing HCC, including imaging modalities and liver biopsy, detect tumors at a relatively advanced stage or are invasive. Non-invasive biomarkers are urgently needed to facilitate screening and early diagnosis of HCC, as well as treatment monitoring and detection of tumor recurrence. Liquid biopsy, the analysis of blood or other body fluids to obtain genetic and epigenetic information, has historically been applied to other types of cancer including breast and prostate cancer. Over the past few decades, liquid biopsy analysis has shed significant insights on genetic and epigenetic aberrations in HCC detectable in peripheral blood. Aberrations in nucleic acids found circulating freely in body fluids or contained within extracellular vesicles such as exosomes or microvesicles show potential clinical utility as non-invasive biomarkers. In this review, we present available literature on cell-free nucleic acids in the diagnosis of HCC.

## INTRODUCTION

Hepatocellular cancer (HCC) has become the second leading cause of cancer deaths worldwide^[1]^. Unfortunately, most cases of HCC are undetected until late stage due to absence of symptoms in early stage HCC, and the lack of sensitive and convenient methods of screening. Previous estimates showed that the one-year survival for HCC in the United States is less than 50%, while the five-year survival is 10%^[2]^. With the advent of potent therapy for chronic hepatitis C virus (HCV) infection, the overall global incidence of HCC may plateau or decrease as a result of decreased HCV-associated HCC, however these gains appear to be threatened by the increasing incidence of nonalcoholic fatty liver disease (NAFLD)-associated HCC and the persistently high levels of hepatitis B virus (HBV)-associated HCC.

Diagnosis of HCC can be made by imaging studies such as multiphasic computed tomography scan or magnetic resonance imaging. However, tissue biopsy remains the gold-standard for HCC diagnosis especially in non-cirrhotic patients or those with nonspecific imaging studies. The risks of biopsy include procedure-related complications such as pain, bleeding, and perforation of adjacent organs, as well as tumor seeding along the needle track and sampling errors resulting in false negative results. Aside from these risks, technological advancements over the past few decades have led to a better understanding of the high heterogeneity and dynamic evolution of HCC tumor cells, and a time- and location-constrained tissue biopsy is inadequate in reflecting these dynamic changes. These shortcomings have led to increased interest in the application of liquid biopsy analysis to HCC. Although the concept of liquid biopsy has been in existence for several decades, the term gained traction in the early 2000’s, with one of its first uses pertaining to the capture of circulating tumor cells (CTCs) for biomarker analysis in breast cancer patients^[3]^.

Liquid biopsy generally refers to the analysis of blood or other body fluids to obtain genetic or epigenetic information which can be applied in screening, diagnosis, prognostication, treatment monitoring or disease recurrence^[4]^. The major advantage of liquid biopsy is non-invasiveness which makes it attractive for frequent analysis to track mutations and other molecular changes over time. The most commonly used HCC serum biomarker is serum alpha-fetoprotein (AFP), together with its fucosylated glycoform (AFP-L3). AFP is normally produced during gestation by the fetal liver and yolk sac, and levels decline rapidly after birth. Regeneration of liver cells leads to AFP production, as can be seen in chronic liver disease and in HCC. Other types of malignancy, for instance testicular or ovarian cancer, can also cause AFP elevation. The AFP-L3 glycoform, named for its ability to bind *Lens culinaris* agglutinin, is a relatively new test developed in 1992 that is more specific for HCC, compared to AFP^[5]^. Serum AFP concentration can be normal even in advanced HCC^[6]^. In two studies of approximately 1800 patients, AFP was found to have about 60% sensitivity and 80% specificity in detecting HCC using a cut off level between 10 to 20 ng/mL^[7,8]^. Higher serum AFP levels are associated with greater specificity and less sensitivity, for instance AFP > 400 ng/mL implies HCC until proven otherwise. However, fewer than 20% of HCC cases have such elevated AFP levels^[9]^.

Serum and plasma biomarkers detectable through liquid biopsy show promise in the early detection of HCC either alone or in combination with AFP. These markers have the potential to be adjunctive or superior to conventional methods of HCC diagnosis. Several of these markers, however, are still in preclinical development and testing and none of them has of yet been recommended for HCC diagnosis. Here, we provide an updated summary of cell-free nucleic acid (cfNA) analysis in the diagnosis HCC, with emphasis on cell-free DNA (cfDNA).

## LIQUID BIOPSY FOR HCC

Liquid biopsy specimens contain genetic information in CTCs or in the form of cfNAs released by apoptotic cells or living cells. cfNA can be found circulating freely in body fluids or are taken up by extracellular vesicles such as exosomes and microvesicles. The various types of cfNA include cfDNA, mRNA (cfRNA), and noncoding RNAs including miRNAs (cfmiRNA). Other noncoding RNAs including long noncoding RNA, small nuclear RNA, small nucleolar RNA, and piwi-interacting RNA may also be present in liquid biopsy specimens and could potentially serve as biomarkers although there are currently very few studies on these subtypes.

The first report of cfNA derived from human peripheral blood was published by Mandel and Metais^[10]^ in 1948, however its significance was not realized until several decades later in 1977 when it was discovered that serum and plasma from cancer patients carry higher concentrations of cfDNA compared to healthy individuals^[11]^. About a decade later, Vasioukhin showed that cfDNA can have cancer characteristics, suggesting that cancer cells can release DNA into peripheral blood^[12]^. This notion was soon confirmed by other investigators^[13,14]^, and cfDNA released by cancer cells into circulation has been subsequently referred to as circulating tumor DNA (ctDNA).

Analysis of plasma and serum cfRNA is limited by the very small quantities present in circulation as well as degradation by ribonuclease (RNase). Incorporation of cfRNA into extracellular vesicles protects them from degradation. Over the past decade, several groups have shown that cfRNA can potentially be applied in HCC detection and monitoring^[15–17]^. A recent study by Xu *et al*.^[18]^ showed that serum mRNA levels of exosomal hnRNPH1 in patients with primarily HBV-associated HCC were significantly higher than in patients with chronic hepatitis B, liver cirrhosis, or healthy control^[18]^. Exosomal hnRNPH1 levels also associated with TNM stage, Child-Pugh classification, portal vein embolism and lymph node metastasis.

Non-coding RNA, especially cfmiRNAs were first demonstrated as a promising biomarker in patients with solid cancers in 2008^[19]^. Since then, there have been several studies on non-coding RNAs in different types of cancer including HCC^[20]^. A recent article mapped the differential expression of non-coding RNAs in normal liver tissue and in various stages of liver disease leading to HCC; each liver phenotype was found to demonstrate a unique RNA signature^[21]^. Induction of exosomal miR-21 and miR-10b in HCC was found to promote cancer cell proliferation and metastasis, potentially serving in prognostication and therapy for HCC^[22]^. Several other cfmiRNAs have been studied, including miR-1^[23]^, miR-16^[24–26]^, and miR-122^[23,27–29]^. An in-depth review of circulating miRNA signatures in HCC is beyond the scope of this article, and the reader is referred to a recent article by Mirzaei *et al*.^[30]^ for further information.

Liquid biopsy analysis in HCC has significantly expanded over the past decade, providing substantial information on different HCC tumors and their microenvironment, and the potential application of such information to disease diagnosis and monitoring.

### Isolation of cell-free DNA in liquid biopsy samples

There are several challenges in the isolation of cfDNA in general and ctDNA in particular, including DNA lysis as a results of blood clotting in collection tubes, DNA contamination during processing or DNA loss during isolation. Thus, the right sample collection tube and optimal processing methods are crucial to the success of isolation and to the accuracy of the sample obtained. A 1 mL volume of blood typically yields 10 ng of cfDNA, and in cancer patients, about 0.01% to 1% of cfDNA comprises ctDNA. Several methods have been used in the isolation of ctDNA, including targeted methods involving polymerase chain reaction (PCR) based on known genetic mutations, for instance digital PCR; bead, emulsion, amplification and magnetics (BEAMing) PCR; and amplification-refractory mutation system-PCR. Alternatively, a variety of untargeted methods can be employed to sequence millions of DNA fragments, including Sanger sequencing and next-generation sequencing techniques such as targeted amplification sequencing or targeted capture sequencing^[31,32]^.

## HCC-ASSOCIATED QUANTITATIVE CHANGES IN CELL-FREE DNA

**Figure 1.**
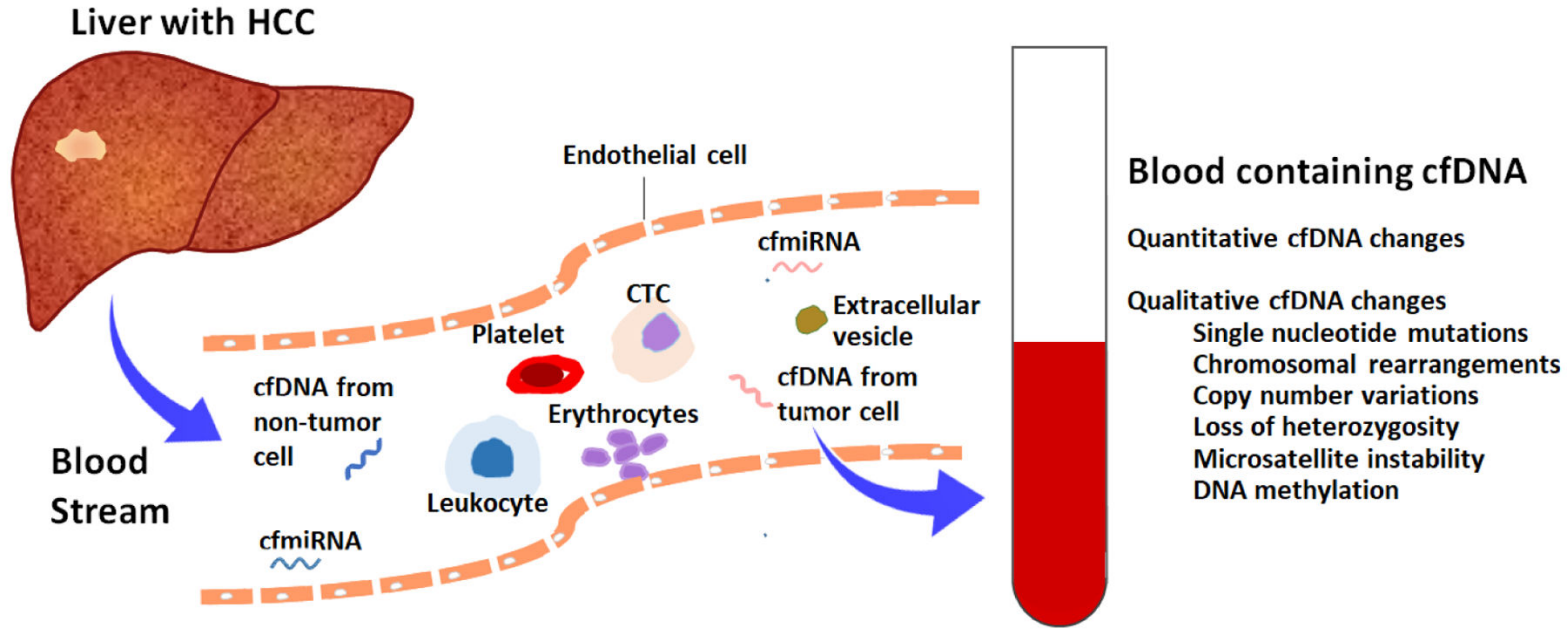
Liquid biopsy for hepatocellular carcinoma. Tumors release a number of molecules into circulation including tumor cells, cell-free DNA, different circulating RNA classes, proteins and extracellular vesicles including exosomes and microvesicles. Cell-free DNA can be isolated from blood or other body fluids and analyzed to determine genetic and epigenetic changes present in circulation which are reflective of changes occurring in tissues, potentially avoiding the need for invasive tissue sampling. cfDNA: cell free DNA; cfmiRNA: cell free microRNA; CTC: circulating tumor cell; HCC: hepatocellular carcinoma

Cancer is associated with both quantitative and qualitative changes in cfDNA detectable by liquid biopsy^[33–37]^ [Figure 1]. In patients with HCC, total cfDNA concentration is significantly higher than in those without HCC^[33,34]^. Although non-specific, cfDNA increase in association with HCC has potential utility in screening for HCC, as well as in monitoring of treatment response and in predicting HCC recurrence^[34,38–40]^. A recent manuscript by Yan *et al*.^[41]^ analyzed cfDNA and AFP levels from 24 patients with HCC and 62 patients with chronic hepatitis B with varying degrees of fibrosis (F0 to F6). Using multivariate analysis, the authors found that age and cfDNA were independent predictors of HCC, while AFP was not an independent predictor. They developed a combination model including cfDNA level, age and AFP, collectively referred to as HCC index, for HCC diagnosis by backward logistic regression analysis. The HCC index showed an area under the receiver operating characteristic curve (AUROC) of 0.98 (95% confidence interval 0.92–1.00), a sensitivity of 87% and specificity of 100% for the diagnosis of HCC at a cutoff value of 0.61^[41]^, proving superior to cfDNA alone or AFP alone in the diagnosis of HCC^[41]^. As shown by Yan *et al*.^[41]^, combination of cfDNA level with other protein or genetic biomarkers holds promise as a liquid-biopsy based clinical tool in the early diagnosis of HCC.

## HCC-ASSOCIATED QUALITATIVE CHANGES IN CELL-FREE DNA

Genetic or epigenetic alterations in cfDNA in association with HCC are detectable by liquid biopsy and are reliable indicators of changes occurring in tumor tissues. In general, these changes are grouped into single nucleotide mutations^[35,42]^, variations in DNA copy number^[35,36]^, chromosomal rearrangements, loss of heterozygosity, microsatellite instability, and changes in methylation pattern^[37]^.

### Single nucleotide mutations

Indepth genomic analysis of HCC tumor tissue to the base pair level has shown that no two tumors carry the same cadre of somatic mutations^[43]^. There is considerable variability in the number of mutations even among patients with advanced stage HCC, as demonstrated by analysis of three patients with advanced HCC which showed 7.2, 15 and 7,910 mutant fragments per 5 mL of plasma^[43]^. Tumor-specific somatic mutations in several genes have been identified in the peripheral blood of HCC patients, including *TP53*^[44]^, *HCK*^[45]^ and *TERT*^[46]^.

The three most frequent somatic mutations in HCC are *TERT* promoter activating mutations which are found in 40%−60% of HCC patients; and the mutually exclusive *TP53* and *CTNNB1* mutations which are found in 30–50% of HCC cases^[47–49]^. Digital droplet PCR to interrogate the single nucleotide mutations *TERT* c.−124C>T, *TP53* c.747G>T (p.R249S), *CTNNB1* c.121A>G (p.T41A) and c.133T>C (p.S45A) in the peripheral blood of patients with predominantly HBV-positive and BCLC stage A showed that 56% of the patients harbored ctDNA containing these mutations^[46]^. *TERT* promoter mutations, however, have been observed in both HCC patients as well as non-HCC cirrhotic patients, suggesting limited utility as a biomarker for HCC^[50–52]^. On the other hand, the *TP53* p.R249S mutation appears specific for HCC and has been identified in plasma, serum and urine samples obtained from cancer patients^[53–57]^. The *TP53* p.R249S mutation is more common in HBV- and aflatoxin-associated HCC, compared to HCC associated with other etiologies. ctDNA in 14 patients with advanced HCC using next generation sequencing showed that somatic *CTNNB1* mutations were the second most common mutation and occurred in 29% of the patients studied^[58]^.

The significant heterogeneity of HCC genetics in association with different etiologies (for instance alcohol related liver disease *vs*. HBV *vs*. HCV *vs*. NAFLD) has posed a major challenge to the development of a universal biomarker panel for detecting HCC. This challenge necessitates the integration of multiple genes and multiple loci within a given gene, as well as combining a vast array of protein and genetic biomarkers. CancerSEEK is a recently developed blood test which detects eight tumor-associated protein biomarkers and mutations (including single base substitutions) in 1933 distinct genomic positions^[59]^. The test was used to query peripheral blood derived from 812 healthy controls and 192 non-metastatic cancers of the breast, colorectum, esophagus, liver, lung, ovary, pancreas and stomach^[59]^. Among 44 patients with HCC, the test a showed 98% sensitivity and 99% specificity in cancer detection. Overall, the test detected five cancer types with sensitivities ranging from 69% to 98%, and with over 99% specificity. The performance of CancerSEEK in differentiating HCC patients from other high risk patients, for instance those with advanced fibrosis or cirrhosis, is yet to be studied.

### Chromosomal rearrangements

Genomic sequencing has identified a number of chromosomal rearrangements in HCC. Ono *et al*.^[60]^ determined cancer-associated genomic rearrangements in HCC tumors through whole-genome sequencing. Subsequently, they validated some of these rearrangements by means of PCR using ctDNA isolated pre-operatively from peripheral blood of HCC patients and primers designed to detect the breakpoints of chromosomal rearrangements seen in tumor tissue. The authors found that pre-operative ctDNA from 7 HCC patients showed several deletions, inversions, tandem duplications and translocations seen in HCC tumor tissue^[60]^. Chromosomal rearrangements can lead to copy number variations and other genetic aberrations, potentially serving as an early noninvasive marker for HCC.

### Copy number variations

Shotgun massively parallel sequencing (MPS) was used to determine tumor-associated copy number variations in the tumor tissue of 4 HCC patients, and in their plasma pre- and post-resection of tumor, compared to 16 healthy controls^[35]^. Characteristic copy number variations in tumor tissue were reflected in pre-resection plasma samples, and were missing almost entirely in post-resection plasma samples. The pre-resection plasma samples detected approximately 10% to 100% of tumor-associated copy number aberrations seen in their corresponding tumor tissue, with detectability of plasma copy number aberrations strongly correlating with plasma ctDNA concentration^[35]^. In another study, MPS analysis of plasma ctDNA size in 90 HCC patients compared to patients with chronic hepatitis B (*n* = 67), hepatitis B-associated cirrhosis (*n* = 36), and healthy controls (*n* = 32) showed that HCC plasma carried high levels of aberrantly short and long DNA^[36]^. The short ctDNA preferentially carried tumor-associated copy number aberrations. Among the 90 HCC patients, 76 (84%) had at least one chromosomal arm-level copy number aberration on chromosomes 1 or 8. In addition, plasma derived from HCC patients contained high levels of mitochondrial DNA albeit much shorter than nuclear DNA^[36]^. The observation that cfDNA in HCC patients are shorter and more fragmented than in patients without liver disease or with non-malignant liver processes has been made by several other investigators^[36,61]^. This observation is worthy of further investigation and may have clinical utility in the diagnosis or monitoring of HCC either alone or in combination with other biomarkers.

### Loss of heterozygosity and microsatellite instability

Pang *et al*.^[62]^ used three high-polymorphic microsatellite markers located on chromosome 8p, D8S277, D8S298 and D8S1771 to examine loss of heterozygosity (LOH) and microsatellite instability. By analyzing plasma cfDNA and tumor tissues from 62 HCC patients, they examined the features of these aberrations in peripheral blood and determined their concordance with tumor tissue. LOH in one or more of the three examined loci was identified in about 58% of patients, occurring at a higher rate in those with metastatic HCC (63%) compared to those with non-metastatic disease (26%)^[62]^. Majority of patients carried microsatellite instability in plasma samples at the same loci as their corresponding HCC tissues, with a concordance rate of about 73%^[63]^. Their findings suggest that LOH and microsatellite alterations may potentially serve in non-invasive diagnosis of HCC, however these alterations generally occur less commonly than the other genetic alterations discussed, and studies are needed to delineate the clinical applicability of these observations.

### Alterations in DNA methylation

DNA methylation, one of the earliest known and well-studied epigenetic modifications, confers changes in chromatin structure, DNA stability and DNA-protein interactions to modify gene expression. Methylation events occur very early in carcinogenesis hence are often detected in precancerous states. To date, several studies have showed that altered DNA methylation at several genes are associated with the initiation and progression of HCC, including *p15* and *p16*^[64]^, *APC*^[65]^, *SPINT2*^[66]^, *SFRP1*^[67]^, *TFP12*^[68]^, *GSTP1*^[69]^ and *RASSF1A*^[70]^. NAFLD-related HCC is associated with hypermethylation of the glycine N-methyltransferase (*GNMT*) promoter, resulting in reduced gene expression^[71]^. Differential DNA hypomethylation has also been seen in HCC. DNA hypomethylation is known to induce several processes leading to transposon activation, chromosomal instability, and the generation of copy number variations. Hypomethylation of repetitive DNA sequences by way of long interspersed nucleotide elements 1 (LINE-1) has been detected in the plasma of patients with HCC^[72]^. Concordance in the methylation profile of several tumor suppressor genes between HCC plasma and tumor tissue has been demonstrated by several studies.

Wong *et al*.^[37]^ showed that 25% of patients with *p15* methylation in tissue also demonstrated methylated *p15* in blood samples, and nearly all patients with *p15* and *p16* methylation in tissues demonstrated methylation abnormalities in blood samples. Importantly, patients with *p15* and *p16* methylation developed HCC metastasis or recurrence after treatment, suggesting that analysis of *p15*/*p16* methylation in cfDNA derived from peripheral blood can serve as a biomarker for predicting the metastasis or recurrence of HCC.

Iyer *et al*.^[65]^ analyzed the tumor methylation profile of several tumor suppressor genes including *APC*, *FHIT* and *E-cadherin* through analysis of plasma and corresponding tumor DNA from 28 HCC patients, as well as plasma DNA from age and sex-matched controls. The analysis showed a statistically significant concordance in methylation profile between plasma and corresponding tumor DNA for all genes analyzed. The concordance for *APC* methylation in plasma DNA *vs*. HCC tumor tissue was almost 82%, with sensitivity and specificity of 78% and 90%. For *FHIT*, the concordance, sensitivity and specificity were all approximately 86%. For *E-cadherin*, concordance was 79%, with sensitivity and specificity of 68% and 100%. As in other studies, *p15* and *p16* methylation patterns were also found to be concordant with sensitivities ranging from 50%−60% and specificities in the 85%−95% range.

*RASSF1A*, a member of the Ras association domain family protein is a tumor suppressor frequently silenced in malignancy by hypermethylation. Serum analysis showed that 90% of HCC patients and 62.5% of HCV patients demonstrate *RASSF1A* hypermethylation, compared to 10% in healthy serum^[73]^.

A biomarker panel based on analysis of a number of genes may serve to better differentiate HCC blood from normal samples, as shown for a combined analysis of the methylation pattern of four genes *APC*, *GSTP1*, *RASSF1A*, and *SFRP1* which showed an AUCROC of 0.933 in identifying HCC from normal samples, compared to 0.800 to 0.881 for the individual genes^[67]^. In another study to evaluate the potential of ctDNA methylation patterns in the diagnosis and prognostication of HCC, Xu *et al*.^[74]^ identified a methylation marker panel differentially enriched in HCC tissue compared to blood leukocytes of healthy individuals. In a training data set of 715 HCC samples and 560 normal samples, the sensitivity and specificity of a 10-marker panel based on methylation patterns were 85.7% and 94.3%, respectively, and a combined prognostic score based on these markers significantly correlated with risk of death^[74]^. These studies suggest that methylation changes characteristic of HCC can be reliably identified in peripheral blood samples and potentially serve as biomarkers for diagnosis and prognostication of HCC.

## CONCLUSION

Liquid biopsy analysis of serum and plasma can reliably detect genetic and epigenetic alterations present in HCC tumor tissue, providing a less invasive alternative to the current gold standard of liver biopsy. Due to the significant heterogeneity of HCC, a single biomarker would lack the requisite sensitivity and specificity for HCC diagnosis, hence a panel consisting of multiple genetic and epigenetic alterations, likely in combination with protein biomarkers, would have the best diagnostic utility. One such test is CancerSEEK, which detects eight tumor-associated protein biomarkers and mutations in 1933 distinct genomic positions, with 98% sensitivity and 99% specificity for HCC detection when tested in 44 HCC patients and 812 controls^[59]^. The authors estimated a cost of about $500 to perform a CancerSEEK analysis^[59]^. Although the test holds promise for diagnosing and monitoring HCC, further studies, including performance of the assay in patients at high risk for HCC such as those with advanced fibrosis or cirrhosis would need to be undertaken. Several other analyses of cfDNA biomarkers either alone or in combination with non-nucleic acid biomarkers for non-invasive diagnosis of HCC are in progress.
